# An Extremely Rare Case of a 24-Year-Old Female Diagnosed With High-Grade Muscle-Invasive Urothelial Carcinoma in the Bladder

**DOI:** 10.7759/cureus.63566

**Published:** 2024-07-01

**Authors:** Baidar Khalabazyane, Mohammad Zuaiter, Roza Salah, Abdirashid Kassim, Atlan Omer

**Affiliations:** 1 Urology, Royal Bournemouth Hospital, Bournemouth, GBR; 2 Urology, North Bristol NHS Trust, Bristol, GBR; 3 Plastic and Reconstructive Surgery, Salisbury NHS Foundation Trust, Bournemouth, GBR; 4 Urology, University Hospitals of Coventry and Warwickshire, Coventry, GBR

**Keywords:** transurethral bladder tumor resection, robot-assisted radical cystectomy, pet scans, transitional cell carcinoma (tcc), bladder cancer in young patients, urothelial cancer of the bladder (ucb)

## Abstract

Bladder cancer most commonly affects older adults. Although extremely rare, it can still be detected in the younger population. Bladder cancer often exhibits distinct behavior in these cases, typically manifesting as a low-grade, non-muscle-invasive disease. We documented a remarkable case involving a 24-year-old female diagnosed with high-grade muscle-invasive bladder cancer. Our report emphasizes the distinctive challenges encountered by clinicians in the journey of diagnosis, treatment, and follow-up of bladder cancer in young patients.

## Introduction

Bladder cancer commonly affects older adults, with 90% of cases diagnosed in individuals over 55 and 80% in those over 65 in the US. On average, bladder cancer is diagnosed at age 73 in the US [1]. While extremely rare, bladder cancer can occur in children and young adults, typically manifesting as a low-grade, non-invasive disease [2]. Research indicates that bladder cancer tends to present differently in younger patients. They typically have smaller, lower-grade tumors (less than 3 cm) and a higher prevalence of papillary urothelial neoplasms with low malignant potential compared to patients over the age of 40. The five-year survival rate is 100% for young patients and 88.1% for older patients [3]. It is well documented that men are diagnosed more commonly with bladder cancer and that women present at a more advanced stage [4].

Despite the rarity of high-grade urothelial cancer in young patients, we present an extremely uncommon case of high-grade muscle-invasive bladder cancer in a young female without identifiable risk factors.

## Case presentation

A 24-year-old female chef, who occasionally smokes cannabis without any other identifiable risk factors, presented in January 2023 with bilateral loin pain, visible haematuria, acute kidney injury (AKI), and sepsis. Blood tests revealed elevated inflammatory markers (C-reactive protein (CRP): 165 mg/L and white blood cell count (WCC): 13 x 10^9^). In addition, hemoglobin dropped to 79 g/dL, and creatinine increased to 165 umol/L from her baseline of 75 umol/L. Following the initial evaluation, further diagnostic studies were pursued.

**Figure 1:**
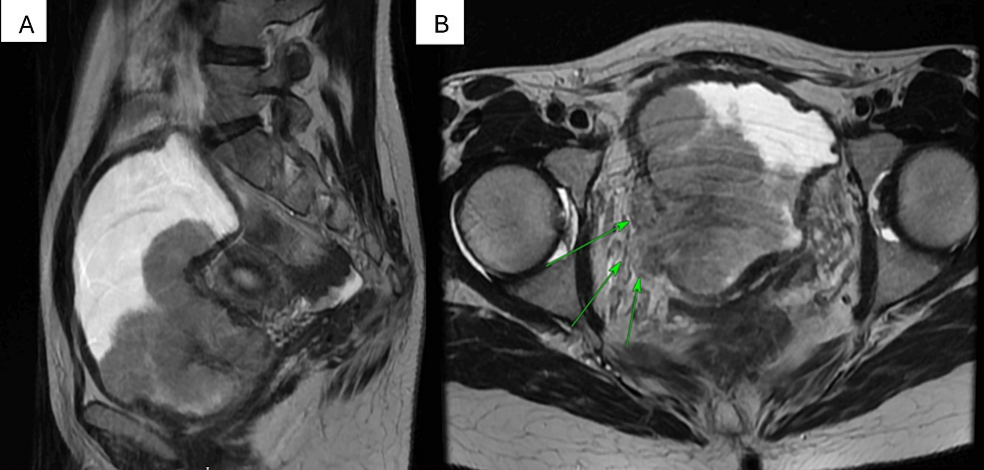
(A) Sagittal section of the MRI abdomen and pelvis. (B) Cross-sectional view of the bladder tumor with evidence of locally advanced bladder cancer extending into the anterior vaginal wall.

**Figure 2:**
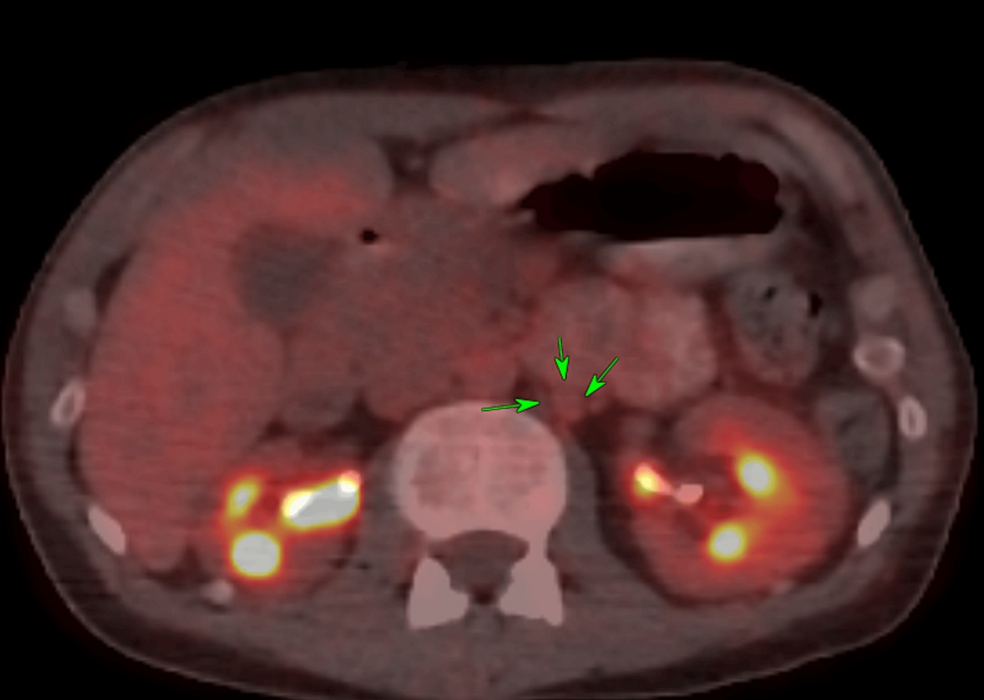
Whole-body PET-CT scan showing minimal activity in the left para-aortic lymph nodes (reactive), with no convincing evidence of disease metastasis.

A CT scan revealed a large bladder mass on the posterior wall measuring approximately 9.2 x 5.3 x 5.6 cm in size, obstructing both ureteral orifices and causing bilateral hydronephrosis. As a result, a bilateral nephrostomy was inserted. To assess the disease's extent, staging scans, including MRI (Figure 1) and PET-CT (Figure 2), were conducted, which confirmed the presence of a large bladder tumor with extra-serosal spread involving the right lateral vaginal wall and both vesicoureteral junctions. However, there was no evidence of distant metastasis. It is worth mentioning that the PET-CT scan showed mild activity in the left para-aortic node, suggestive of reactivity, without evidence of distant lymphadenopathy or metastatic disease. The overall final clinical staging was T4.

Afterward, she underwent a transurethral resection of a bladder tumor (TURBT), revealing a high-grade, solid tumor occupying most of the bladder. The resection was incomplete, aimed primarily at histological diagnosis rather than staging. Histopathology confirmed papillary urothelial cancer G3 and at least PTa.

The case was discussed in the multidisciplinary team (MDT) meeting, and given the extravesical extension of the tumor, neo-adjuvant chemotherapy was recommended. Considering her young age, she was referred to the Centre of Reproductive Medicine for egg retrieval before chemotherapy. However, due to the lengthy pre-retrieval process, which would have delayed the cancer treatment, the patient chose not to proceed with this option. She received four cycles of neoadjuvant chemotherapy (gemcitabine and cisplatin) without any complications.

**Figure 3:**
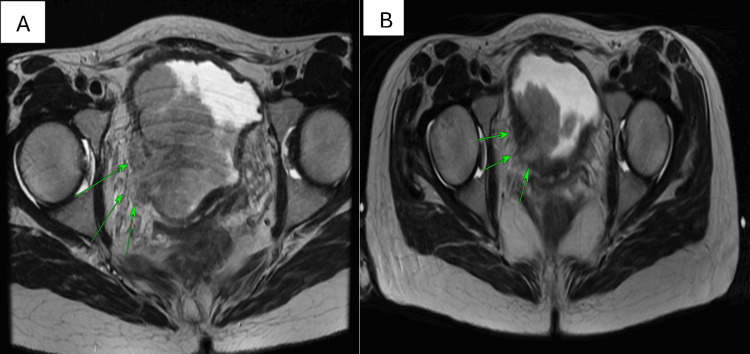
Before (A) and after (B) neo-adjuvant chemotherapy.

After the neoadjuvant treatment, the patient had re-staging MRI scans, revealing a good response to the neo-adjuvant chemotherapy (Figure 3).

**Figure 4:**
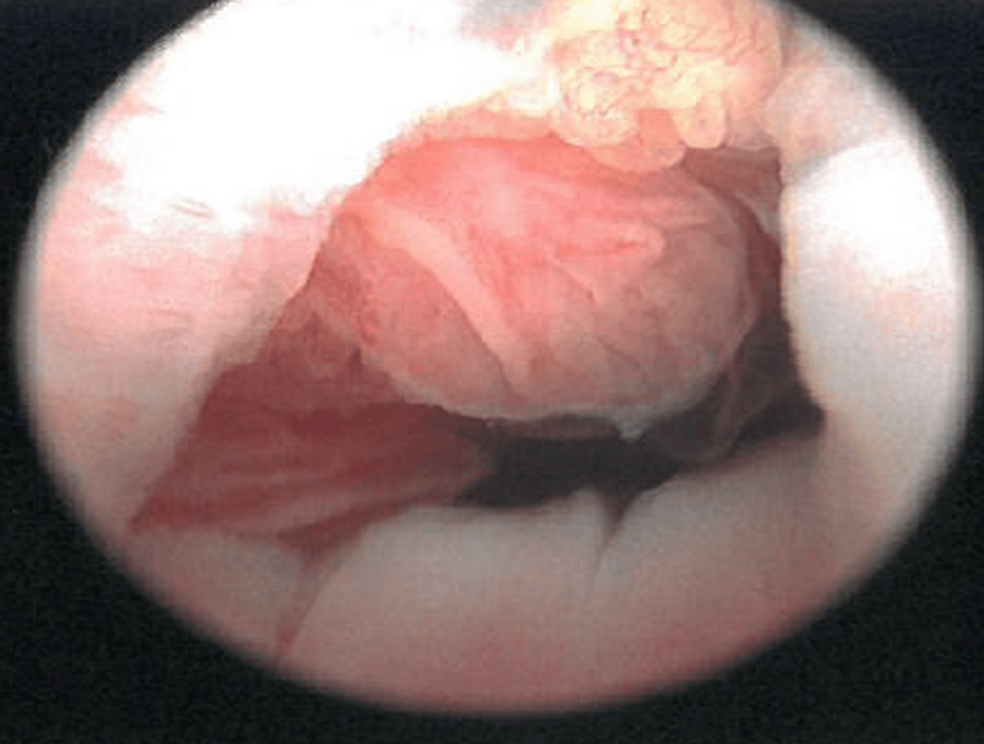
Cystoscopic view of the tumor with macroscopic extension and involvement of the bladder neck.

The patient was counseled on various surgical management options, and after discussion, she chose radical cystectomy with neobladder formation. Therefore, she underwent a bladder neck biopsy and examination under anesthesia (EUA), which revealed bladder neck invasion by the tumor (Figure 4).

Two separate bladder neck biopsies were taken, revealing high-grade papillary TCC, G3 pTa. Subsequently, her case was reviewed again in the MDT meeting, and it was decided that she was not a candidate for a neo-bladder formation.

Eventually, she underwent robot-assisted radical cystectomy with ileal conduit formation, bilateral extended pelvic lymphadenectomy, and preservation of sexual organs.

**Figure 5:**
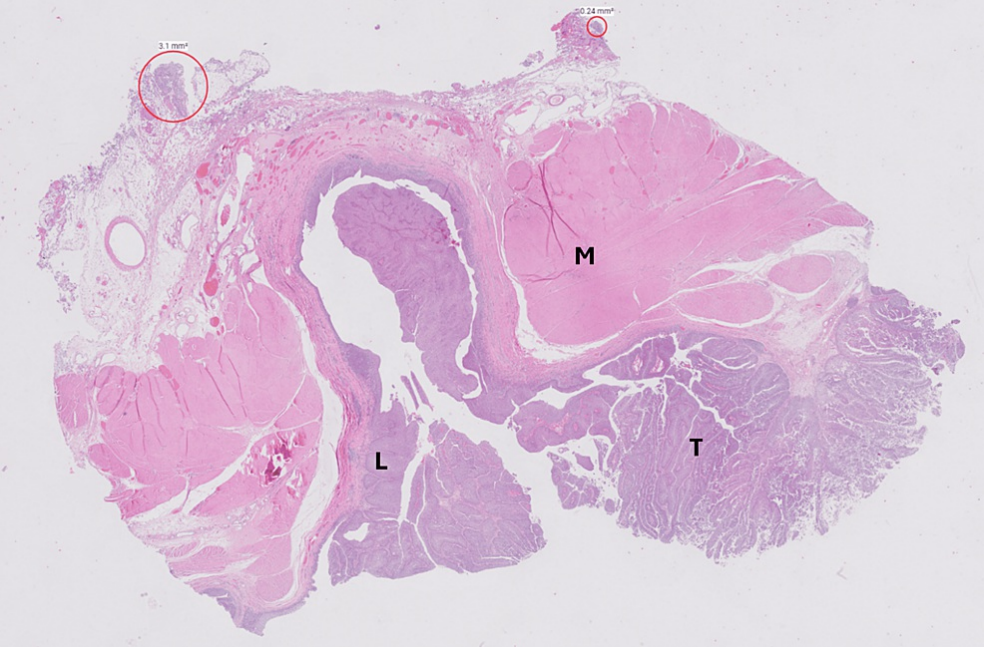
Histopathological examination of the bladder showing tumour invading into the lamina propria while sparing the muscularis propria, represented by letters. M: muscularis propria, L: lamina propria, T: tumor, hematoxylin and eosin stain

The surgical approach was selected for its minimally invasive nature and potential for enhanced patient outcomes. A total of 34 lymph nodes were submitted for histopathological examination, and all showed no signs of invasion. In addition, the surgical margins were negative. The final histopathology report revealed T1 high-grade urothelial carcinoma with associated carcinoma in situ (CIS), resulting in a final stage of ypT1 ypN0 (Figure 5).

It is crucial to exclude germline mutations, particularly in young patients. Immunohistochemical evidence commonly shows overexpression of the p53 gene product in bladder cancer among young patients [4]. Microsatellite alterations are frequent early events and might have prognostic significance in bladder cancers arising at a young age [5]. However, our patient's final report showed no evidence of microsatellite instability.

The patient had a swift and uneventful post-operative recovery, as the patient was discharged on the sixth postoperative day. Regular follow-up CT chest abdomen and pelvis were scheduled every six months to monitor the patient for potential disease recurrence.

## Discussion

Bladder cancer is a prevalent urinary system malignancy, primarily linked to cigarette smoking and certain occupational or chemical exposures. However, it is rare in young adults and presents unique diagnostic and management challenges, significantly impacting patients' psychosocial well-being. This group often hesitates to adhere to strict follow-up [6], as seen in our patient who missed several follow-up appointments because she was in a denial phase and afraid of undergoing a major operation.

Young patients with bladder cancer have unique characteristics and higher overall and cancer-specific survival rates compared to elderly patients [7]. However, clinicians should be aware that patients under 40 years of age present with higher-grade and larger (>3 cm) tumors and are more likely to experience tumor recurrence [8].

Furthermore, young bladder cancer patients have a lower male-to-female ratio and a lower likelihood of advanced stages and high-grade cancers at the initial diagnosis. Tumors in young patients are typically less multifocal at diagnosis. In addition, young patients have a lower recurrence rate and longer recurrence interval than older patients [9].

It is imperative to screen for genetic mutations in this age group, as immunohistochemical overexpression of the p53 gene product is common and microsatellite alterations are frequently early events in young bladder cancer cases [5].

Neoadjuvant chemotherapy improves overall survival and causes pathological downstaging in muscle-invasive bladder cancer [10]. Initially staged as T4 on imaging, our patient was down-staged to ypT1 ypN0 after neoadjuvant chemotherapy treatment.

Prognosis is critical for younger bladder cancer patients, necessitating curative treatments that improve the quality of life. Fertility preservation strategies should be thoroughly discussed with all young patients.

The decision to perform a radical cystectomy for this patient was driven by the high-grade nature of the tumor and muscle invasion on imaging and her young age. The use of robotic-assisted surgery allowed for precise and minimally invasive tumor resection. Remarkably, the patient's recovery time was fast, and she was discharged on the sixth postoperative day.

## Conclusions

This case report presents a rare instance of high-grade muscle-invasive bladder cancer in a 24-year-old female without significant risk factors. The case highlights the critical importance of thorough evaluation of hematuria in young patients, regardless of risk profile. It demonstrates the complex interplay between oncological management and quality-of-life considerations, particularly regarding fertility preservation and urinary diversion options. The successful use of neoadjuvant chemotherapy leading to pathological downstaging, followed by robotic-assisted radical cystectomy with sexual organ preservation, exemplifies the potential benefits of a tailored, multidisciplinary approach in young patients.
